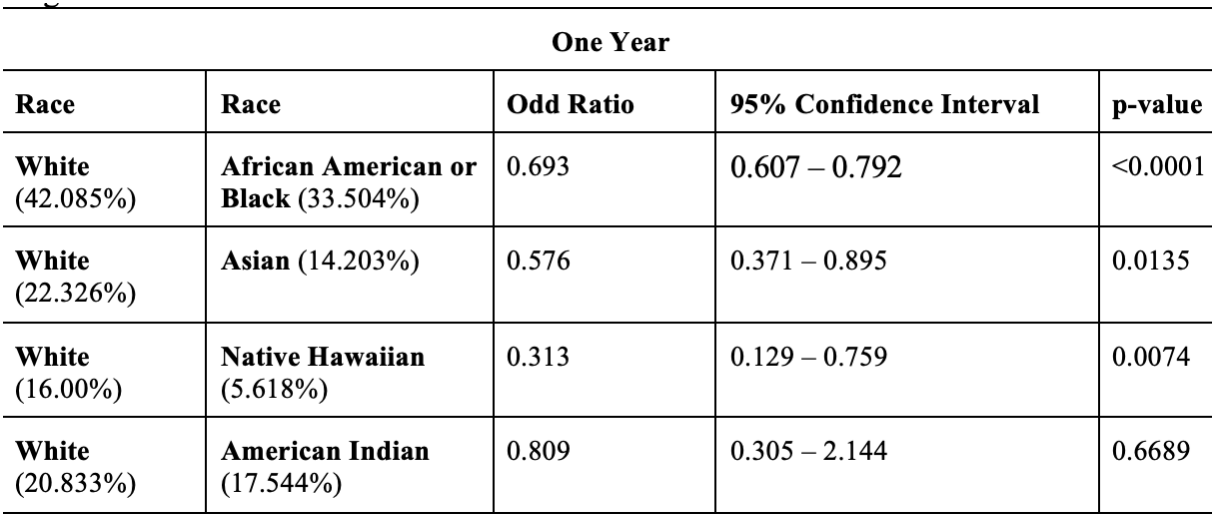# 102 A Lower Prescription of Opioids in Treating of Chronic Pain in Black Burn Patients

**DOI:** 10.1093/jbcr/iraf019.102

**Published:** 2025-04-01

**Authors:** Joshua Lewis, Bethel Desta, Gengi Kleto, Blancheneige Beohon, Mbinui Ghogomu, Raven Hollis, George Golovko, Juquan Song

**Affiliations:** University of Texas Medical Branch; University of Texas Medical Branch; University of Texas Medical Branch; University of Texas Medical Branch; University of Texas Medical Branch; University of Texas Medical Branch; University of Texas Medical Branch; University of Texas Medical Branch

## Abstract

**Introduction:**

Chronic pain is a common and debilitating outcome for many burn patients, necessitating pain management strategies. Despite the potential for addiction and abuse, opioids are frequently prescribed for managing severe and chronic pain. Previous studies have highlighted that Black patients are less likely to receive an opioid prescription compared to their white counterparts. However, the other race related difference has been not fully studied. This study investigates whether racial disparities in opioid prescriptions for chronic pain persist in burn patients.

**Methods:**

Using the TriNetX database, cohorts were identified based on ICD-10 codes for adult patients aged 18 years or older diagnosed with chronic pain over the year and previous burn injuries. Propensity score matching for burn severity, age, and ethnicity was performed to balance the cohorts. Opioid prescription rates among racial groups were examined using univariate regression models, calculating odds ratios (ORs) with statistical significance set at p< 0.05.

**Results:**

Among 32,167 burn patients who developed chronic pain and was prescribed, 63.66% (n=20,478) were White, 17.80% (n=5,726) were Black or African American, 2.57% (n=827) were Asian, 1.09% (n=351) were Native Hawaiian, and 0.60% (n=193) were American Indian, highlighting racial variation in the prevalence of chronic pain post-burn injury. The odds of receiving an opioid prescription to treat chronic pain in burn patients were significantly lower for African American or Black patients (OR: 0.693, p < 0.0001), Asian patients (OR: 0.576, p = 0.0135), Native Hawaiian patients (OR: 0.313, p = 0.0074), and patients of Other Race (OR: 0.641, p = 0.0081) compared to White patients. American Indian patients did not show a significant difference (OR: 0.809, p = 0.6689).

**Conclusions:**

This study reveals significant racial disparities in opioid prescriptions for chronic pain management in burn patients. These findings underscore the urgent need for targeted interventions and policy changes to ensure equitable pain management and address these disparities, promoting comprehensive care for all racial groups.

**Applicability of Research to Practice:**

The findings of this study are highly applicable to clinical practice, particularly in the management of chronic pain among burn patients. By highlighting significant racial disparities in opioid prescription patterns, healthcare providers are encouraged to reevaluate their pain management strategies to ensure that they are equitable across all racial and ethnic groups. This research underscores the importance of developing standardized guidelines that reduce unconscious bias and promote fair treatment for minority populations. Additionally, it offers valuable insight for policymakers to address structural barriers in pain management, leading to more comprehensive and culturally competent care protocols that can improve health outcomes in underserved communities.

**Funding for the Study:**

This research was supported by a Clinical and Translational Science Award (UL1 TR001439) from the National Center for Advancing Translational Sciences at the National Institutes of Health (NIH). The content is solely the responsibility of the authors and does not necessarily represent the official views of the NIH.